# The impact of respiratory viruses on lung health after preterm birth

**DOI:** 10.1080/20018525.2018.1487214

**Published:** 2018-08-01

**Authors:** Nada Townsi, Ingrid A. Laing, Graham L. Hall, Shannon J. Simpson

**Affiliations:** a Children’s Lung Health, Telethon Kids Institute, Perth, Australia; b Division Paediatrics, University of Western Australia, Perth, Australia; c Department of Higher Education, Ministry of Education, Riyadh, Saudi Arabia; d School of Biomedical Sciences, University of Western Australia, Perth, Australia; e School of Physiotherapy and Exercise Science, Curtin University, Perth, Australia; f Centre of Child Health Research, University of Western, Perth, Australia

**Keywords:** Viruses, respiratory infection, preterm, lung, infants, bronchopulmonary dysplasia

## Abstract

Children born preterm, less than 37 weeks’ gestation, are at increased risk of viral respiratory infections and associated complications both during their initial birth hospitalisation and in their first years following discharge. This increased burden of viral respiratory infections is likely to have long term implications for lung health and function in individuals born preterm, particularly those with bronchopulmonary dysplasia. Several hypotheses have been put forward to explain the association between early life viral respiratory infection and development of suboptimal lung health and function later in life following preterm birth. Although preterm infants with diminished lung function, particularly small airways, might be particularly susceptible to asthma and wheezing disorders following viral infection, there is evidence that respiratory viruses can activate number of inflammatory and airway re-modelling pathways. Therefore, the aim of this review is to highlight the perinatal and early life risk factors that may contribute to increased susceptibility to viral respiratory infections among preterm infants during early life and to understand how respiratory viral infection may influence the development of abnormal lung health and function later in life.

## Preterm birth and bronchopulmonary dysplasia: an immature system

Every year, more than 11% of children are born preterm; equating to an estimated 15 million of the global population. Complications of prematurity underlie more than half of all neonatal deaths worldwide, such that over one million infants die each year due to complications of preterm birth [1]. For those who survive the neonatal period, many face a lifetime of ongoing health problems with a substantially increased risk of significant respiratory morbidity that persists through life [2–4]. The risk and severity of lifelong morbidity and mortality are accentuated by the level of prematurity [5,6], birth weight [6] and the presence of bronchopulmonary dysplasia (BPD) [7].

Chronic lung disease of prematurity, or BPD, was first described by Northway over 50 years ago as a severe pulmonary disease primarily caused by aggressive mechanical ventilation and prolonged exposure to high oxygen concentration in an era before the widespread use of antenatal steroid and postnatal surfactant [8]. The significant advances in neonatal care over the last decades have resulted in increased survival at lower gestational age and consequently a profound change in the clinical and pathological characteristics of BPD; leading to the emergence of ‘new’ BPD [9–11]. New BPD is classified in infants born <32 weeks gestation, based on the requirement for supplemental oxygen for at least 28 days after birth, with severity assessed at 36 weeks post-menstrual age [11]. Unlike the older description of progressive fibroproliferative disease [8], new BPD is characterised by premature interruption of alveolar and micro-vascular maturation leading to fewer, immature, larger and more simplified alveoli along with dysmorphic pulmonary vascular development [10, 12].

**Figure 1.**
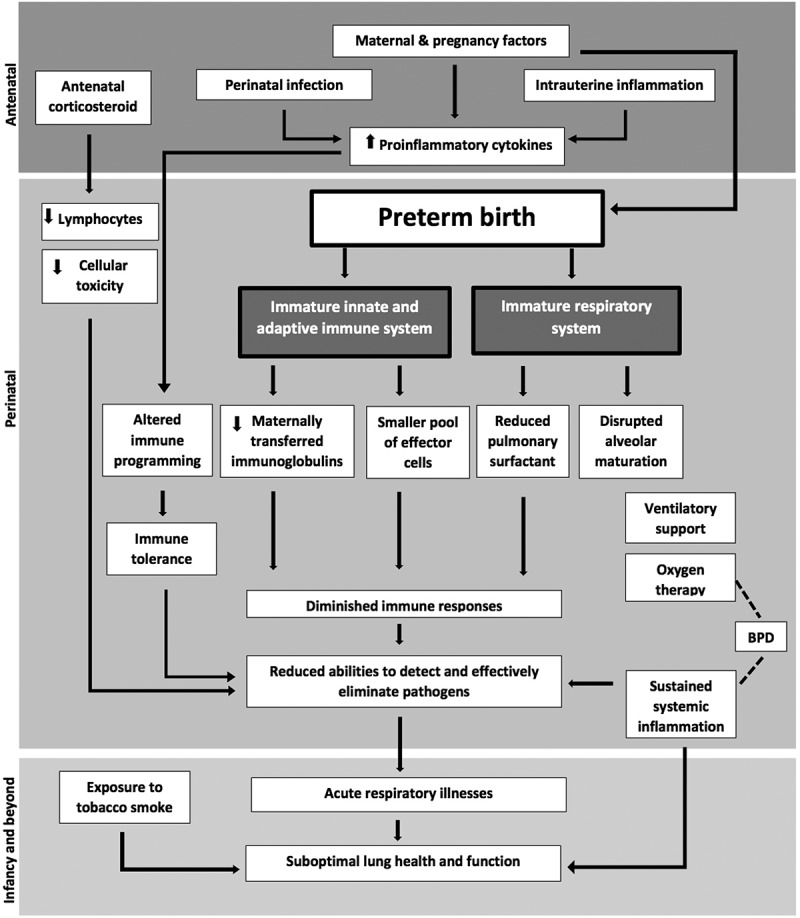
Schematic diagram showing pathways whereby exposure to perinatal and early postnatal influences associated with preterm birth may result in diminished immune responses and increased susceptibility to acute respiratory infection and thereby, increased vulnerability to suboptimal lung health and function later in life.

Alongside the structural immaturity associated with preterm birth and subsequent development of BPD, many preterm infants have insufficient production and composition of pulmonary surfactant [13]. Surfactant is crucial for maintaining alveolar functional stability through lowering surface tension at the air–liquid interface and, thereby preventing lung collapse at end-expiration [13]. The biological role of surfactant, however, in modulating and maintaining pulmonary host defence against infection and inflammation has become increasingly recognised [14], with several studies reporting increased risk of infection and inflammation in term-born children and adults with surfactant deficiency or inactivation (Figure 1) [15,16].

In addition to pulmonary structural and functional immaturity, preterm birth is also associated with developmental immaturity of innate and adaptive immune systems as well as a significant lack in the functional interaction between the two systems as outlined in Figure 1. During early life, all infants rely heavily upon maternally transferred antibodies as well as components of the innate immune system as a first line of defence and protection against invading pathogens due to the relative immaturity of the pathogen-specific adaptive immunity, which gradually develops after birth [17–19]. However, these defences against pathogens are severely compromised in preterm infants [20]. Features of immaturity of innate immunity following preterm birth including a smaller pool of effector cells (neutrophils, monocytes, natural killer cells and antigen presenting cells) and lower levels of inflammatory cytokines have been reported by several studies [18,21,22]. In addition, the levels of antigen specific immunoglobulins are substantially reduced in preterm infants, since these antibodies are largely transferred across the placenta during the third trimester, particularly after the 32nd week of gestation [23]. Preterm birth is also associated with limited production and function of antimicrobial proteins, peptidases and soluble cytokines [22,24,25].

Other perinatal and neonatal factors associated with preterm birth have also been associated with altered immune programming during early life, which may result in lifelong adverse immune consequences among survivor of preterm birth [18]. Exposure to perinatal infection and intrauterine inflammation have been associated with increased production of inflammatory cytokines and premature activation of the immune system which can skew immune responses toward a hyporesponsive (tolerant) phenotype and result in long-term immune programming effects [26,27]. Similarly, exposure to other non-infectious influences including oxidative stress and shear forces generated from supplemental oxygen and mechanical ventilation have also been associated with increased secretion of pro-inflammatory cytokines resulting in sustained systematic inflammation and which can further compromise immune responses [18]. Diminished ability to detect and eliminate viruses and other pathogens due to reduction in the number of circulating lymphocytes have also observed in preterm infants following antenatal corticosteroid administration, a well-established practise to improve foetal lung maturation and reduce pulmonary complications when preterm labour are suspected (Figure 1) [28].

The disruption to lung development and diminished immune responses place survivors of preterm birth, at increased risk of severe respiratory infection during early life. Early-life exposure to such pathogens is likely to have lifelong respiratory consequences. Indeed, even in healthy term-born infants, hospitalisation with acute respiratory viruses in early life is associated with increased rates of childhood asthma, wheezing disorders and suboptimal lung function [29–31]. While preterm infants are often labelled as asthmatic, the underlying mechanisms of prematurity associated wheeze are likely to be different [32] although the long-term implications of exposure to acute respiratory viruses during early life remains to be elucidated in the preterm population. The following sections will review what is known about the burden and consequences of early-life infection with respiratory viruses among preterm infants.

## Viral infection in preterm infants during the birth hospitalisation

The role of immature respiratory and immune systems has a critical impact on susceptibility to nosocomial infections and infection-related complications during the birth hospitalisation [33]. Although bacterial outbreak has long been the main focus of nosocomial infection during the neonatal period, previous studies have demonstrated that viral outbreaks can contribute to an equivalent level of mortality (6.4% vs. 7.17% respectively) [33,34]. The role of viruses in nosocomial infection has become increasingly recognised, with several studies showing that 20 to 50% of very preterm infants, born at ≤ 32 weeks’ gestation, acquired a nosocomial infection with a respiratory virus during their birth hospitalisation, often in the absence of clinical indicators of respiratory illnesses [35–37]. The detection of asymptomatic respiratory viruses was associated with deleterious outcomes, such as prolonged hospitalisation, increased number of clinical deterioration events as well as a greater requirement and longer duration of supplemental oxygen and ventilatory support [33,34,36,37]. In addition, the risk of developing a BPD diagnosis was more than two-times higher among infants with detected respiratory viruses during their birth hospitalisation [36,37]. Collectively, such findings may advocate for more comprehensive routine testing for respiratory viruses in preterm infants during the neonatal hospitalisation

## Viral infection requiring readmission in early life

Following the neonatal hospitalisation, the overall risk of re-hospitalisation with an acute respiratory infection remains at least three times higher during the first year of life compared to infants born at term [2,3,38,39]. The frequency of re-hospitalisation during the first year of life among preterm infants ranges from 6 to 50% [5,40–42] depending on the cohort studied. Young chronological age is the main predictor of infection-related morbidity among preterm infants, with studies reporting increased risk of infection, healthcare utilisation and hospital readmissions during the first 6 months of life [39,43,44].

Reduced gestational age is another well-known risk factor for respiratory infection-related re-hospitalisations during early life [39], with community-based studies reporting increasing cost, risk and severity of infection as gestational age decreases [5,40,45]. In one series, preterm infants born at less than 25 weeks’ gestation had more than twice the frequency of re-hospitalisation due to acute respiratory infections and more than twice the length of hospital admission compared to those born in the late preterm period (31% and 12 days vs. 13% and 5 days, respectively) [40]. More recent analysis from a large population based, data-linkage study reported a 12% increase in the overall frequency of infection-related hospitalisation during childhood for each week reduction in gestational age [6]. In addition to reduced gestational age, low birth weight, although at least partly inter-dependent with gestational age, has also been attributed to a 19% increase in the proportion of infants hospitalised with acute respiratory infection for each 500 g reduction in birth weight [6,41].

Preterm infants with certain coexisting morbidities, including BPD, congenital heart disease or chronic oxygen dependency are particularly at risk for severe infections and infection-related hospitalisation during early life [39]. Preterm infants with BPD have an increased frequency of health care utilisation and hospital admissions due to acute respiratory infections compared to those without BPD [2,38,46]. Approximately, 50 to 73% of preterm infants with BPD were re-hospitalised at least once with an acute respiratory infection during the first 3 years of life, which is nearly twice the frequency of re-hospitalisation among those without BPD [7,47,48]. The risk of re-hospitalisation due to acute respiratory infection is significantly higher among infants with BPD who remain oxygen dependent after discharge during the first 3 years of life, compared to those who are not oxygen dependent at discharge (70% vs. 58% respectively; *P* < 0.001) [48]. Similarly, the risk of re-hospitalisation due to acute respiratory infections is more than 4-fold higher among preterm infants with congenital heart disease compared to otherwise healthy preterm infants [49].

Several other factors have been attributed to the substantial vulnerability to respiratory infection and infection-related hospitalisation in preterm infants. Previous genetic studies have demonstrated a significant association between the inherited polymorphisms in several immunological and surfactant protein genes and the incidence of severe viral infection and level of infection-related morbidity among preterm infants [50,51]. Also, pre-existing impairments in lung function have also been attributed to increased susceptibility to severe viral infection and infection-related morbidity among preterm infants, particularly during early life [52–54].

### Early-life infection with RSV

Nearly, all children become infected with RSV during the first 2 years of life, yet only 1% requires hospitalisation [55]. However, incidence of RSV-related hospitalisation among preterm infants ranges from 10 to 19% during the first year of life [55–60]. The risk of RSV-related hospitalisation is significantly influenced by the neonatal diagnosis of BPD, with studies reporting a 2- to 7-fold increased risk of hospitalisation during the first 2 years of life among preterm infants with BPD compared to those without BPD [60,61], and more than 7-fold increase compared to healthy term-born infants [62]. In addition, RSV-related morbidity is higher among preterm infants, particularly those with BPD, with a greater risk of increased clinical deterioration events, higher therapeutic intervention scores, prolonged hospitalisation and greater requirement for admission to the neonatal intensive care unit (NICU) during RSV-related hospitalisation compared to term-born infants [43,59,63]. One particular study of clinical outcomes during RSV-related hospitalisation showed that preterm infants had significantly longer hospital stays (17 vs. 8 days; *P* < 0.001) as well as more frequent (41.4 vs. 12.6%) and longer stays in the NICU for invasive respiratory support (13 vs. 6 days; *P *< 0.001), compared to term-born infants [64].

Increased clinical care for preterm infants hospitalised with RSV translates to increased morbidity, healthcare utilisation and costs of care for respiratory illnesses later in life [65,66]. Early-life hospitalisation with RSV infection among preterm infants has been associated with more than a 2-fold increase in the mean number of subsequent hospitalisations during the 4 years following the initial infection (1.28 vs. 2.96; *P *< 0.001), when compared with non RSV-related hospitalisation [67]. In addition, hospitalisation with RSV infection during infancy is associated with increased odds of subsequent hospitalisations with non-respiratory conditions including anaemia, anorexia and fever. Of particular importance, RSV-related hospitalisation in preterm infants has been associated with more than 5-fold increase in the risk of overall death within the first 4 years following the initial infection (Odds ratio = 5.5; 95% CI, 4.6–6.6; *P* = .001), compared to non RSV-related hospitalisation [67].

Early-life exposure to severe RSV infection has also been identified as a main contributor in the development of suboptimal lung health and function later in life, with several studies reporting increased risks of late-onset or persistent wheezing phenotypes, asthma diagnosis, and impaired lung function in adolescents and young adults [29–31]. Even though the majority of the data on the long-lasting impact of RSV have been based on studies of healthy term-born infants, limited studies have demonstrated increased risk of asthma [68], recurrent wheeze [34,69,70] and lung function impairments [66] in preterm children with a history of RSV-related hospitalisation during infancy. Early-life hospitalisation with RSV infection in preterm infants has been associated with more than twice the risk of ongoing respiratory morbidity, with wheezing rates ranging from 20.7 to 42.8%, 1–2 years following RSV-related hospitalisation compared to 4.1 to 23% following non-RSV related hospitalisation [43]. The risk of asthma, recurrent wheeze and long-lasting impairments in lung function following severe RSV-related respiratory illness during infancy is more pronounced among preterm infants with BPD [66,71] and those born at a lower gestational age [68]. Although most studies demonstrating persistent respiratory morbidity following RSV-related respiratory illness have focused on hospitalised infants, a single study has demonstrated persistent airway resistance at 1 year of age in preterm infants following mild RSV infection that did not require hospitalisation [72].

Although it is possible that the immature lungs in preterm infants might be particularly susceptible to long-term damage following severe RSV-related respiratory illness during early life [66,73], RSV infection during such a critical period of lung growth and development can also result in chronic alterations and remodelling of the developing airways independent of pre-existing neonatal factors [50,72]. The underlying mechanisms through which RSV could promote the development of suboptimal lung health and function later in life are yet to be fully elucidated.

Several mechanistic pathways have been hypothesised to describe the potential role of severe RSV-related respiratory illnesses in the pathogenesis of asthma and wheezing disorders later in life, including persistent activation of atypical immune responses and alterations to the structure and function of the developing airway [50,72,74]. Mouse models have also demonstrated the ability of RSV to induce and maintain prolonged inflammatory responses leading to significant damage to the airway epithelium, as well as chronic airway remodelling and persistent airway hyperresponsiveness following the initial infection [75–77]. Alterations of the local production of immunoregulatory cytokine, particularly interleukin-10, have also been identified as an important contributor to the subsequent development of recurrent wheeze and persistent airway hyperresponsiveness following RSV infection [78–80]. The ability to alter local immune responses has also been proposed as a key mechanism, with some evidence describing the ability of RSV to redirect immune cells and maintain a low level of replication in immunologically privileged sites within the lung (possibly involving neuronal or lymphoid cells) in order to avoid recognition by the immune system [76,77,81]. Such alterations in immune responses sequentially result in latency and chronic persistence of the RSV genome within the lung for several weeks following the initial infection, with studies reporting significant association between the detected level of RSV within the lung and the level of both airway hyperresponsiveness and persistent airway inflammation [76,77].

Protection against RSV using palivizumab, a humanised monoclonal antibody for RSV prevention, can result in significant reductions in both acute and chronic morbidity following RSV-related illnesses in preterm infants, with previous studies reporting up to a 50% reduction in the frequency of hospitalisation and a 60 to 80% reduction in the relative risk of recurrent wheezing disorders during the first years of life [82–84]. However, the use of palivizumab is currently restricted to high-risk infants, as the American Academy of Pediatrics only recommends immunoprophylaxis with palivizumab during the first year of life for infants born at <29 week’s gestation with coexisting morbidities including BPD or hemodynamically unstable cardiovascular disease [85]. Such restricted use of palivizumab has been largely related to the high cost of this treatment (estimated cost for a single course approximately $US 4458 per child), as well as the uncertainty about cost-effectiveness, with recent studies reporting limited or no effect of palivizumab among otherwise healthy preterm infants born at or after 29 weeks’ gestation [86–88]. To date, there is no effective acute treatment for RSV, even in high risk groups such as preterm infants. Current recommendations advocate supportive measures only such as respiratory support and hydration [89]. Nevertheless, new RSV treatments such as vaccines and therapeutic agents are currently under development [90]. The successful implementation of these agents will depend on a comprehensive assessment of the burden and risk factors for RSV illnesses and morbidity, in preterm infants both in the community as well as those admitted to hospitals, so that vulnerable infants can be targeted for RSV prevention interventions.

### Early-life infection with RV

Studies of the burden and impact of non-RSV respiratory viral illnesses on the subsequent development of chronic respiratory morbidity in preterm infants have been limited. Rhinovirus (RV) is a major cause of acute respiratory illnesses and wheezing episodes in infants and young children and has been associated with an increased health burden, particularly among preterm infants [91–94]. Although RV infections have long been thought to be limited to the common cold and mild self-limiting upper respiratory illnesses, the application of improved viral molecular detection methods has substantially improved understating of the epidemiology and the clinical significance of RV, particularly among high risk children [94–98]. In a 2-year prospective study, RV was detected in 41% of all episodes of acute respiratory illnesses and 33% of related hospitalisations during the first year of life in a cohort of preterm infants [99]. The risk and severity of RV-related respiratory illnesses are considerably elevated among preterm infants with BPD, with a more than 5-fold increased risk of RV-related hospitalisation among infants with BPD compared to non-BPD infants [99].

Similar to RSV, RV-related respiratory illness during early life has also been associated with a high risk of suboptimal lung health and function later in life [100–102]. Insight on the association between RV-related respiratory illnesses and the risk of future asthma originated from the Childhood Origins of Asthma Study, a prospective birth cohort of term-born infants at high risk of asthma and atopy [103]. The prevalence of asthma at 6 years of age was more than three times higher in children who wheezed in the first three years of life during RV-related respiratory illnesses compared to RSV-related respiratory illnesses (Odds ratio = 9.8 vs. 2.6; *p* < 0.05); even though the majority of these illnesses did not require hospitalisation [104]. Another finding from this cohort suggested that the occurrence of RV-related wheezing illness in the first three years of life is associated with significant airflow limitation and lung function impairment at 5 to 8 years of age [102]. Hospitalisation with RV-related respiratory illness has been associated with a more than 3-fold increase in the rate of recurrent wheeze among healthy term-born infants during the following year [105]. Similarly, hospital presentation and/or admission with an RV-related wheezing illness in childhood is associated with a 3-fold increased risk of subsequent hospitalisation for another respiratory illnesses [106] as well as a more than 4-fold increased risk of subsequent asthma [100].

The long-lasting impact of early-life RV-related respiratory illnesses among preterm infants remains to be elucidated. Furthermore, despite strong epidemiological evidence supporting the role of RV in the pathogenesis of asthma, airway reactivity and airflow limitation, the underlying mechanisms are unclear. Although damage to the developing airways and altered immune responses are thought to be the main mechanisms through which RV could induce asthma and airway hyperreactivity later in life [107,108], others have suggested that early-life infection with RV might be the first indication of the pre-existing tendency in some children to develop asthma, including among preterm infants [109,110]. The ability of RV to induce prolonged and exaggerated inflammatory responses after the initial infection has been described as a potential contributor to the development of chronic respiratory morbidity among preterm infants, with limited data demonstrating increased airway secretion of inflammatory cytokines and remodelling molecules during acute RV infection, particularly among those with BPD [108,111]. The dysregulated immune response to RV infection was associated longitudinally with more severe respiratory morbidity, increased hospitalisation and requirement for intensive care admission during the first 2 years of life [108].

The synergistic interaction between viral infections, most commonly RV, and sensitization to aeroallergens during early life has also been linked to the risk of developing asthma later in life in term-born children [101,103.104,112]. However, these interactions are less evident among preterm-born children who show low levels of exhaled nitric oxide and little evidence of eosinophilic inflammation compared to term-born asthmatic children [114,115]. The occurrence of atopy and allergic sensitization is less frequent following preterm birth, which might be related to altered immune programming during early life and the shift toward a more tolerant immunophenotype [18]. Thus, different pathophysiological mechanisms, other than allergic sensitization, are proposed to underlie the increased risk of asthma and wheezing disorders following preterm birth. This hypothesis has been supported by studies describing the lack of association between allergic sensitization, viral infection and the risk of asthma and diminished lung function later in life among preterm-born children [116–118].

### Early-life infection with previously under-recognised and newly emerging viruses

Influenza-related respiratory infection has long been considered as a leading cause of morbidity, hospitalisations and mortality among the elderly. However, equivalent levels of morbidity, complications, hospitalisations and costs of care have been attributed to influenza-related respiratory illnesses in young children [119–121]. The burden of influenza is inversely related to age, with 50% of influenza-related hospitalisations occurring in infants younger than 6 months of age [120,122]. Data from a wide population-based study of children under 5 years who were admitted to hospital or presented to outpatient clinic with an acute respiratory illness, over five influenza seasons, showed that 35% of hospitalised children and 7% of outpatient children had laboratory-confirmed influenza, despite the extended vaccination recommendations in the population studied [123]. The long-term impact of influenza-related respiratory illnesses during early life is largely unknown, particularly among preterm infants.

The significance of several previously under-recognised and newly emerging viruses is yet to be investigated. Human metapneumovirus (hMPV) was first isolated in 2001 from young children with a spectrum of clinical disease similar to that of RSV [124] and has since been considered as a major pathogen for acute respiratory illnesses and recurrent wheeze in young children, predominantly during the first year of life [125–127]. The risk of hospitalisation following hMPV infection is more common among children with underlying comorbidities including preterm birth [128]. Acute and chronic morbidity following hMPV-related hospitalisation is high among preterm infants, manifesting with prolonged hospitalisation, increased need for respiratory support compared to term children [129] and abnormal lung function at 1 year of age following initial infection [73].

Human bocavirus (hBoV) was first identified in 2005 from the nasopharyngeal aspirate of patients with unresolved respiratory infection [130]. HBoV has also been associated with acute respiratory illnesses, predominantly among young children [131–133], and has been suggested as a potential cause of acute respiratory illnesses and recurrent wheezing episodes across several studies of preterm infants and children [134–136].

In summary, knowledge on the long-term impact of viral infections during early life, particularly among preterm infants remains limited. The vast improvements in molecular detection techniques during the past few decades have shed light on several previously under-recognised circulating respiratory viruses as well as improved the characterisation of newly emerging viruses. However, the disease burden and long-term clinical and functional impact of a variety of early-life viral infections among preterm infants, particularly those with BPD, has not been comprehensively assessed.

Despite mounting clinical and experimental evidence, the potential association of viral-related acute respiratory illnesses during early life and the subsequent development of suboptimal lung health and function is still debated. It remains unclear whether early-life infection with respiratory viruses may induce long-term deficits in the structure and function of the respiratory system through damaging the airways, and altering immune responses or whether these illnesses simply unmask a pre-existing tendency for chronic respiratory morbidity in children at risk, including preterm infants. Although it is likely that the significant immaturity of the structure and function of the respiratory system in preterm infants contributes to chronic respiratory morbidity later in life, viral-induced acute respiratory illnesses during a critical period of lung growth and development may also be an important stimulus for persistent airway remodelling. Additional studies are needed to characterise and assess the consequences of early-life infection with respiratory viruses among preterm infants – both symptomatic and asymptomatic. Identifying the mechanistic role of respiratory viruses in the development of chronic respiratory morbidity is equally important and may aid the design of more targeted therapeutic strategies to reduce virus-induced pulmonary morbidity, and potentially halt the progression of, or even prevent the development of irreversible airway disease.
